# Personalized online information search and visualization

**DOI:** 10.1186/1472-6947-5-6

**Published:** 2005-03-14

**Authors:** Dongquan Chen, Helmuth F Orthner, Susan M Sell

**Affiliations:** 1 Biostatistics and Bioinformatics Unit, Comprehensive Cancer Center. University of Alabama at Birmingham (UAB). Birmingham, Alabama, USA; 2 Department of Health Services Administration, School of Health Related Professions (SHRP), UAB. Birmingham, Alabama, USA; 3 Department of Nutrition Sciences, SHRP, UAB. Birmingham, Alabama, USA

## Abstract

**Background:**

The rapid growth of online publications such as the Medline and other sources raises the questions how to get the relevant information efficiently. It is important, for a bench scientist, e.g., to monitor related publications constantly. It is also important, for a clinician, e.g., to access the patient records anywhere and anytime. Although time-consuming, this kind of searching procedure is usually similar and simple. Likely, it involves a search engine and a visualization interface. Different words or combination reflects different research topics. The objective of this study is to automate this tedious procedure by recording those words/terms in a database and online sources, and use the information for an automated search and retrieval. The retrieved information will be available anytime and anywhere through a secure web server.

**Results:**

We developed such a database that stored searching terms, journals and et al., and implement a piece of software for searching the medical subject heading-indexed sources such as the Medline and other online sources automatically. The returned information were stored locally, as is, on a server and visible through a Web-based interface. The search was performed daily or otherwise scheduled and the users logon to the website anytime without typing any words. The system has potentials to retrieve similarly from non-medical subject heading-indexed literature or a privileged information source such as a clinical information system. The issues such as security, presentation and visualization of the retrieved information were thus addressed. One of the presentation issues such as wireless access was also experimented. A user survey showed that the personalized online searches saved time and increased and relevancy. Handheld devices could also be used to access the stored information but less satisfactory.

**Conclusion:**

The Web-searching software or similar system has potential to be an efficient tool for both bench scientists and clinicians for their daily information needs.

## Background

The rapid growth of publications available in the Medline and other sources raises the question how to search efficiently while maintaining acceptable relevancy. Without the assistance from domain experts such as medical professionals or librarians, retrieving relevant information from the Internet remains a difficult task. For bench scientists, it is important to monitor articles in their fields constantly on a weekly if not daily basis. For a clinician, it is important to access the patient records anywhere and anytime.

These searching procedures often involve relatively simple and similar procedures. For example, they go to certain websites such as the Medline, type in certain words or terms, and then search accordingly using the search engine of that site. Different words or combination reflect different research topics. The returned results were viewed and discarded. The similar search may be repeated next time by typing the same words/terms again.

The objective of this study is to automate this tedious procedure by recording and parsing theses words and websites into a database and to search automatically using software. The person who records the words could be a librarian or a person familiar with medical subject heading (MeSH). The advantage of using MeSH terms is to increase both recalls (sensitivity) and relevancy (specificity) since Medline is indexed through the MeSH. The software could be software agent that automatically performs certain procedure such as searching the Web using words from the database. The advantage of using software is to save time, especially when the search is conducted during night time. It is usually faster due to less congested network traffic. The returned search results were stored locally on a server and could be updated regularly. We have developed such a system and implemented in our Cancer Center website. We emphasized the security issues due to the potential of the system in retrieving information from privileged sources such as a patient record system.

A Web-based user satisfaction survey showed that the personalized information search increased both efficiency and relevancy. We concluded that the Web-searching agent or similar system has potential to be an efficient tool for both research scientists and clinicians to get desired information automatically from various sources.

## Implementation

### System architecture and the agent software

The system was created as a Web-based search, storage, and presentation system with both wired and wireless components. We include the wireless component due to ever increasing popularity of personal digital assistant (PDA) such as a pocket PC, Palm pilot, or other handheld devices in personal information access. The wired component was based on the Ethernet that links the users and the web server. The wireless component included several wireless LANs (WLAN) with Access Point (AP) was centrally controlled by an Access Control Server (ACS, Cisco Company) for Authentication, Authorization and Accounting (AAA). All wireless clients had Extensible Authentication Protocol (EAP)-enabled as described before [1]. The scheduling software agent called Schedule Wizards was installed and programmed to use the stored user preference such as words, websites, and journals in the database to search the various information sources automatically.

### Database, coding, schedules, automatic search, storage and visualization

The database was developed based on Microsoft (MS) Access. User-defined scripts were inserted for using the agent software. Web-based administrative interface for information presentation, and preference updates were created by using MS FrontPage. Hyper Text Markup Language (HTML), active server pages (ASP) with that links web interface with the database server through open database connectivity (ODBC), VBscript, and Structured Query Language (SQL) computer languages were applied for preference collection and storage. The Boolean expressions "AND", "OR" and "NOT" were applied to filter the articles, focus the search, and increase relevancy.

The returned search results were stored locally, as is in a html file, in a MS Internet Information Server (IIS) and updated daily. Each user preference has option to have 4 or more topics that each includes four MeSH terms, a website, a update schedule, and as many journals, which are coded using their International Standard Serial Number (ISSN). The four terms are linked by two "AND" Boolean expressions and one "NOT" Boolean expression. The users log onto the website to view the updated search results without typing any words. The system has potentials to retrieve similarly from non-MeSH-indexed literature or a privileged information source such as a clinical information system where user account information are needed for authentication. The security is thus addressed by applying access control strategy coupled with user account management.

### Online survey and data analysis

An online survey form was created and the survey conducted during the testing period. The questions had been designed to collect user information, to survey the user satisfaction and to determine the efficiency and effectiveness of the system. Although not the focus of the study, questions related to recall (sensitivity) and precision (specificity) were also included. Statistical analysis using paired t-test assuming equal variance was conducted to compare time spent before and after using the system. The evaluation of the relevancy is based on the user-defined criteria. Theoretically, both the relevancy and recall using MeSH may be higher than the search based on unmodified words, since parsing from regular words into MeSH terms could increase recall and precision from the MeSH-indexed Medline.

## Results

### System and database development

**Figure 1:**
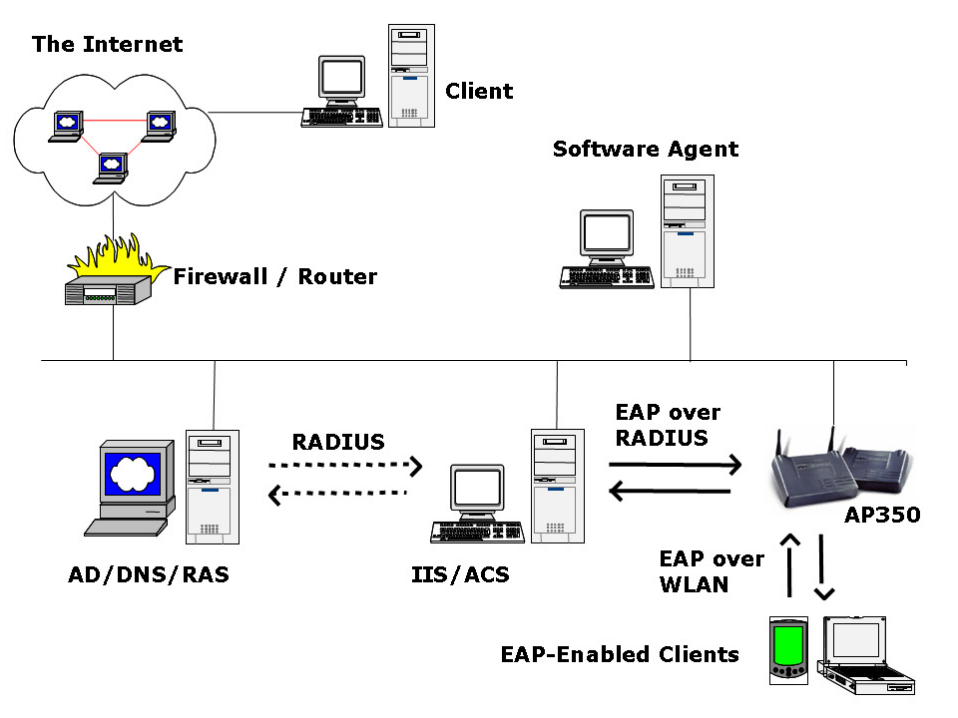
**Web-based, wireless information access and security management. **The AP 350 was configured to use ACS for authentication of the EAP-enabled wireless devices over the WLAN. The IIS offers presentation interfaces for the stored information. A W2K Server running AD was used to mimic a NAS to communicate with ACS through RADIUS protocol. The NAS has enabled RAS for the ACS.

**Figure 2:**
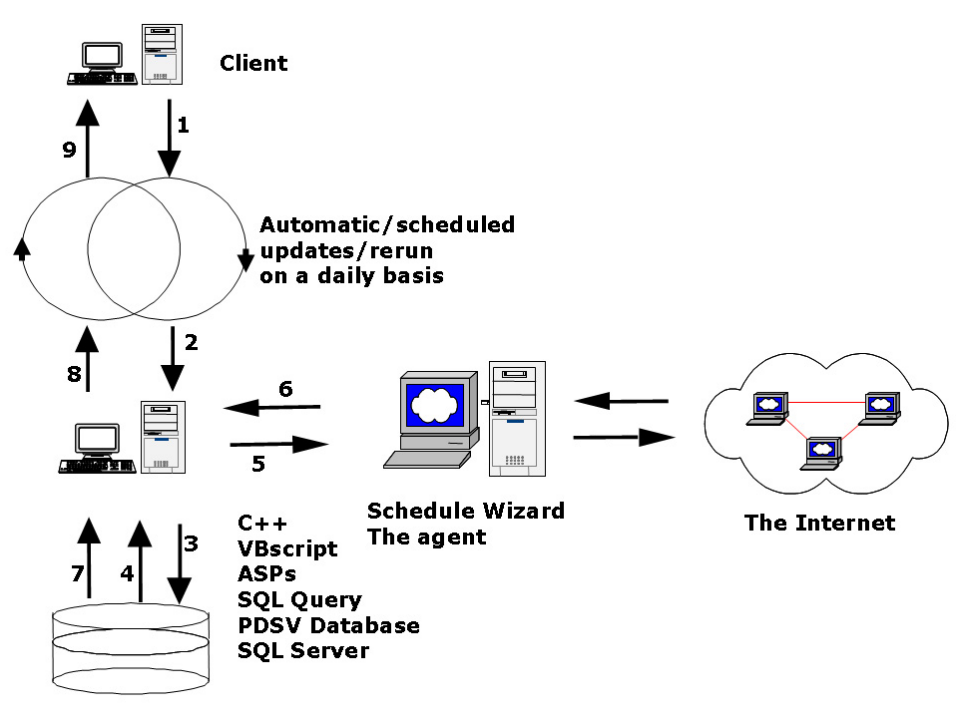
**The automatic search procedure by the Medline Agent. **The automatic search includes the recording, parsing and storage of user preferences (steps 1–3), code-generation and search by the software (steps 4–5), storage (step 6) and presentation (steps 7–9) or the retrieved information. User preferences were stored in the database and will later be parsed into the executable code. The software performed search automatically according to the designated schedule and preference such as website, terms and sources. The retrieved information was stored within the IIS and presented over the Web.

The system adopted a client and server architecture to centralize search, storage and management as shown in Figure 1. The software agent runs on the server using the data for the database to search the Medline. The Active Director (AD) of a Windows 2000 (W2K) Server is used as a Network Access Server (NAS) that negotiates with ACS for the AAA services. The database includes various tables to record information about user preference (words/MeSH terms, website, search schedule, et al). All the information can be updated over the Web using an online preference form. The overall search contains multiple steps as shown in the Figure 2. The system administrator discusses with end users for setting up user accounts and recording their preferences. A librarian or any person familiar with MeSH can do the same. A Web-based form is available for the user to view current preferences and update the preference over the Web. The recorded words are then matched to MeSH terms. The modified preference is then parsed into the code that the software agent will apply to perform the search accordingly and automatically. The search is conducted automatically according to the user preference mostly during the night. This is to take advantage of the less-congested network traffic during the night time.

### The Web interfaces for visualization

**Figure 3:**
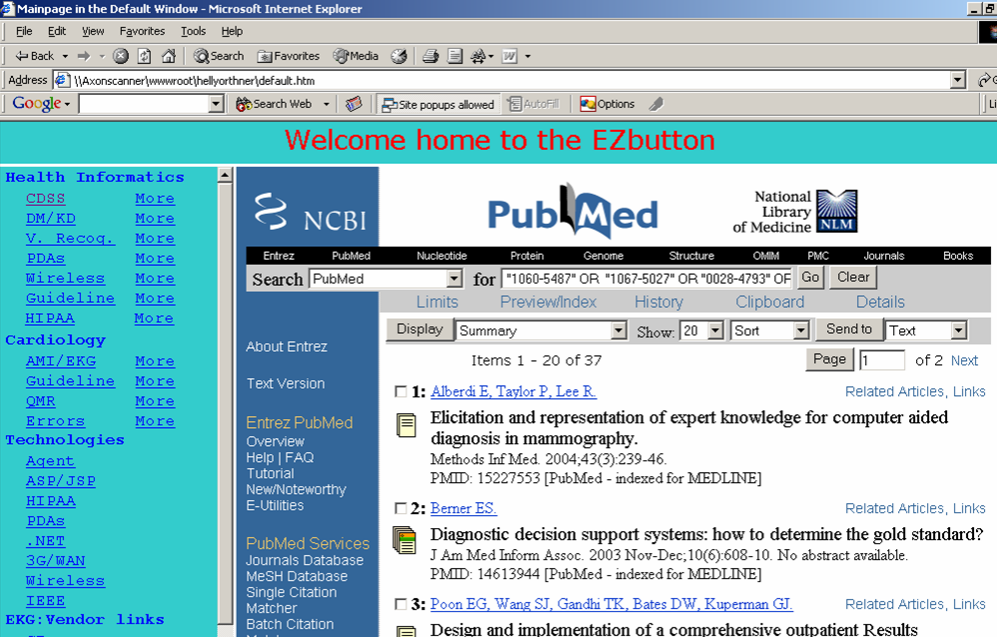
**The Web-interface for clients/users. **The automatic search was conducted as scheduled based on the user preferences. Individual users logon to their own interface to see publications from the Medline (upper section in left panel) and from the other sources (lower section of the left panel). The right panel show the sources when linkages on the left panel are clicked. The listed publications reflects the updated search results. Clicking on the linkage on the right panel leads to the abstracts just like you search the Medline regularly.

The system was implemented in a testing Web server and Web access interface designed for individual user. Figure 3 showed one of the typical Web interface for interested topics. Clicking on the related linkage left column leads to the PubMed search page, as shown in the right column. Theoretically, the user could have as many topics but we limited it to around four for the testing purposes. The users do not have to type in any words since those words have been recorded, optimized through MeSH website , and used in generating the updated linkages to the publications.

**Figure 4:**
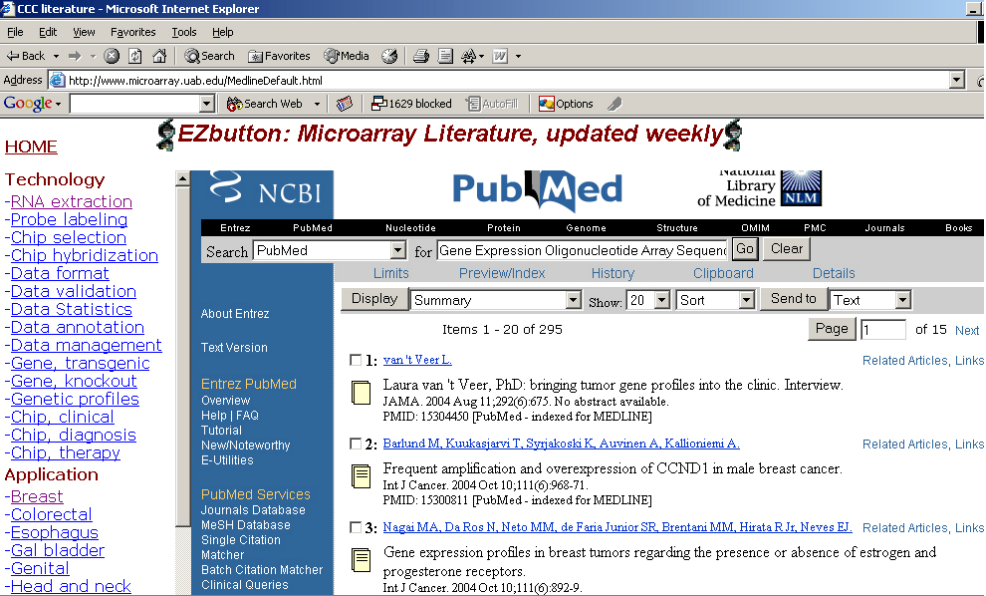
**The Web-interface for microarray-related publications. **The automatic search was conducted as scheduled based on the predetermined MeSH terms. The left panel includes the microarray-related technology (upper section) and clinical applications (lower section), which are different sites studied. The right column shows the interface of Entrez PubMed. The listed publications reflects the updated search results. Clicking on the linkage in the right panel leads to the abstracts just like you search the Medline regularly.

The system was also implemented in the Cancer Center Microarray Shared Facility web site  to retrieve and update regularly the Microarray-related publications, as show in the Figure 4. The disease sites such as Breast and Colorectal et al were selected since the related clinical trials are currently conducted in the Center. Investigators view the updated publications without typing a word. The searching terms has been mapped through MeSH tool in Entrez PubMed website in order to increase the sensitivity and specificity.

### Coding of personal agent to use the database

The Schedule Wizard was the scheduling software we used to manage the scheduled procedures such as daily backup and searching job using Web browsers such as MS Internet Explorer (IE). The software was programmed to turn on at nighttimes to search and retrieve relevant information from the Medline. Various search engines over the Web were used to search the user predetermined topics. The returned search results were stored as a HTML file and saved locally on a server within a MS IIS. The scheduled search and retrieval were performed according the user-predefined schedule on a daily, weekly or monthly base. The visualization interfaces were linked to those stored results.

Part of a sample code for a single search (one topic) is listed below:

START "C:\PROGRAMS\Internet Explorer\IEXPLORE.EXE" "C:\PROGRAMS\Internet Explorer"

WAIT 3

KEYS [Ctrl-TAB][ENTER]

IFTIME 300

WAIT 3

KEYS [Ctrl-A]

KEYS "0028-4793" OR "0098-7484" OR "1532-0464" OR "0007-1447" OR "0026-1270" OR "1475-3898" OR "0003-4819" AND Radiology Information Systems AND Computer Storage Devices AND Computer Communication Networks [ENTER]

WAIT 3

KEYS [Alt-f] [a]

KEYS C:\Inetpub\wwwroot\Agent\All in one\RISprefered.htm [ENTER]

WAIT 1

KEYS [TAB] [ENTER]

WAIT 3

END

KEYS [Alt-f] [c]

The above example is to search and retrieve information regarding "radiology information system", "storage devices" and "networking". The preferred journals are indicated as ISSN codes. The line 1 is to start MS IE followed by 3 second for the browser to be fully loaded. Line 3 is to direct the agent to the PubMed  Entrez searching page with the default searching filed. Line a) to line k) conducts the search and downloads the searching results in a folder called "All in one" and named "RISprefered.htm" within the IIS. The "IF" statement reinforces the rule that the search will be terminated with "END" if time lasts more than 300 seconds. This is to ensure timely closure of searching window in order not to interfere with the next scheduled searching task. Many search procedures could be concatenated forming a single search task, which could be executed all at once. The stored search results are immediately available for viewing through the browser after an authorized user logs on to the system and is directed to the stored HTML file in IIS.

### Effectiveness and efficiency of the Medline search by user satisfaction survey

The main goal of our system was to save time for bench scientists and clinicians. We tested the feasibility of an automated search process based on user preferences. Although not the focus of the study, the question related to recall and precision were asked in the survey. Similar to the sensitivity and specificity, **recall **refers to the proportion of relevant documents in the collection retrieved by a query [2-5], and **precision **the proportion of the relevant documents returned by the query [2]. From the online survey, the system was rated as useful and easy to use, as shown in the Table 1. Most users did not know MeSH concept well and intend or recommend using the MeSH and the system. The time spent to retrieve similar amount of information decreased from 2.2 hours to 1 hour. Almost 70% information retrieved was considered relevant. Among the relevant information, one third was considered new to them. All these suggest that the system or similar products may potentially be useful tools to improve search efficiency and relevancy as well. The keys may hinge on individualization and specification. User information also suggests that search process is time-consuming process and may prompt more use of MeSH terms in the future.

**Table 1:** The system saves time and improves online search efficiency (n = 35)

**Questions**	**Criteria**	**Scale**	**Mean ± SD**
The system			
	useful	1 for not at all and 9 very useful	7.5 ± 1.4
	easy to use	1 for very difficult to 9 very easy	6.6 ± 2.3
	will use it regularly	1 for not and 9 certainly will	6.5 ± 2.2
	recommend using it	1 for not and 9 highly	7.5 ± 2.0
Efficiency			
	time prior to using it	0.5, 1.0, 1.5, ...4.0 hrs/wk	2.2 ± 1.3
	time after using it	0.5, 1.0, 1.5, ...4.0 hrs/wk	1.0 ± 1.2*
Relevancy			
	relevant articles	10, 20, ..., 90%	69.3 ± 19.4
	new among the relevant	10, 20, ..., 90%	33.6 ± 15.7
Users			
	know MeSH before	1 for not at all and 9 for expert	3.2 ± 2.0
	new to computer	1 for new and 9 for expert.	6.3 ± 2.4
	reasons to try it		
		too busy to search	74%
		too time consuming	43%
		too many irrelevant reports	17%
		frustrated when searching	11%

*p < 0.001 using paired t-test assuming equal variances as compared with hours spent weekly prior to using system.

### System stability, wireless access and security

The stability and reliability of the system were tested with tasks scheduled 5-minutes apart. The average time for one search and retrieval task, which includes many searching topics concatenated, was 5–10 minutes. It will serve more than 8,000 users if a dedicated computer is used to conduct search on a monthly basis. Among all scheduled tasks, most (99%) were successfully accomplished during 5 months of testing period, except those during power outages and network downtime (data not shown).

The wireless LAN managed wireless clients from a centralized ACS as reported before [1] and shown in the Figure 1. We chose to include the wireless component due to the increasing popularity of the technology. The security concern has to be addressed first. It has been reported that through open-air clear text transmission of Wired Equivalent Privacy (WEP) keys and Media Access Control (MAC) addresses increased vulnerability [6]. A W2K Server running AD and Domain Name System (DNS) were thus implemented and used to enhance the security. AD and DNS mimic a Network Access Server that was needed for the wireless clients to communicate with ACS through Remote Access Dial-in User Service (RADIUS) and EAP-based protocol. No apparent weakness has been reported yet. The NAS has enabled a Remote Access Service (RAS) for the ACS. The ACS takes advantage of Windows security management features as applied in AP management. The centralized control of all wireless access points and clients enhanced the secure transmission of the stored information over the WLAN.

## Discussion

### Standardized procedure and automated online search

Our study is in an attempt to standardize and to automate a time-consuming but relatively simple and similar searching procedure, not trying to do traditionally considered Information Retrieval (IR) [7-10] text mining [11] or Automatic knowledge extraction [12]. To reduce the time spent on the Medline search, different approaches such as stored procedures, filtering and librarian assistances have been applied. Locally-stored procedures, however, is less accessible than stored information, since the procedures have to be stored locally and executed again. Web-based search system may provide user-friendlier presentation and accessibility.

Software agent is a robot software program that carries out set of operations on behalf of a user with some degree of independence or autonomy. Functionally, an software agent can be as simple as an autonomous software programs that assists the user's daily routine such as reading electronic mail and maintaining a calendar. The Multi-Agent Retrieval Vagabond on Information Networks (MARVIN) has been developed for search from clinical systems [13]. The system is not, however, tailored to individual need. It is possible to automatically search, retrieve, and present information by the joined efforts of both end users and system administrators and others [14]. The personalized automatic searching and presentation system will help bench scientist and clinicians in their daily information acquisition.

### Individualized search may enhance both relevancy and efficiency

Individualized online retrieval from the MeSH-indexed Medline or other sources such as a local medical information system was expected by many clinicians to improve their decision-making [15]. Many commercial products intend to personalize the user preference. The low specificity of a profession-based rather than individual-based filtering system limits their capability to maintain precision for a research scientist or other medical professional, e.g., an oncologist of B cell lymphomas, let along when personal criteria of relevancy may change over time [16]. The real challenge is how to extract individual needs into controlled vocabulary such as MeSH in order to achieve highest possible relevancy and efficiency [14]. Understanding the user's need and knowledge of the searching area may result in higher quality search from, e.g., MeSH-index sources such as Medline.

### Mobile devices and security management for privileged information

The widely used mobile devices such as Pocket PC or Palm Pilots are getting relatively inexpensive and may be applied in daily information access [17]. The use of wireless access to broadband services may mean that even full motion video applications could be supported over long distance [18]. We foresee a wider use of wireless devices for daily information needs.

The security concerns for accessing retrieved information through both wired and wireless network still prevent the healthcare organization from deploying them. One of the approaches to minimize the vulnerability is to control the remote and/or wireless clients' access through RADIUS and EAP-enabled AP management [1]. Another approach is to use digital certificate and secure web servers [19,20]. In addition, personal-identifiable data from patient should be guarded with highest possible security measure [21], especially because of the Health Care Insurance Portability and Accountability Act (HIPAA). Combined efforts of technical, organizational and behavioral approaches are needed to guard the stored information and at the same time to make authorized access easier.

### The limitation of the system and future directions

There are several limitations related to the study. First, individualization of the searching strategy involved the interaction between system administrator and end users for their preferences thus limit the automatic nature of the system. Further study to automate the procedure based on the online preference modification is needed to expand the user pool. Promotion of MeSH awareness is another way to increase its use and enhance recall and relevancy for MeSH-indexed sources. Secondly, WLAN provided access to the stored information within the campus. Real time Internet access including Virtual Private Network (VPN) and digital certificates should be further tested although our preliminary results indicated the possibilities (data not shown). Thirdly, most current users were recruited among users within Medical Informatics field including teachers and students or research labs in Biology. Expansion into other fields will further demonstrate the usefulness of the system. Lastly, pocket PC does not seem to be a good choice for viewing images due to its small screen and low speed wireless connection. Other handheld devices with bigger screen and higher visibility such as tablet should be tested for files like images and electrocardiogram.

## Conclusion

The primary goal of this study is to test the feasibility of automated search from various sources, especially from online literature sources such as the MeSH-indexed Medline and access the stored information through a Web interface. The survey analysis based on user-defined criteria or relevancy revealed a significant reduction of time spent on the Medline search while maintaining a relatively high relevancy. This might due to individualized and MeSH-based searching strategy. To further enhance the effectiveness and efficiency, more users are to be recruited. Security concerns need to be thoroughly addressed before implementing wireless access or accessing clinical information.

## Availability and requirement

• Project name: personal agent for information search and retrieval.

• Project home page: .

• Operating system(s): Web-based system, platform independent.

• Programming language: C++, JavaScript and html.

• Other requirements: none.

• License: free to access.

• Any restrictions to use by non-academics: none.

## List of abbreviations

AAA: Authentication, Authorization and Accounting.

AD: active directory.

ASP: active server pages.

AP: access point.

DNS: domain name system.

EAP: Extensible Authentication Protocol.

HTML: Hyper Text Markup Language.

IIS: internet information server.

MAC: Media Access Control.

MeSH: medical subject heading.

NAS: Network Access Server.

ODBC: open database connectivity.

PDA: personal digital assistant.

PDSV: personal domain specific vocabulary.

RADIUS: Remote Access Dial-in User Service.

RAS: Remote Access Service.

SQL: Structured Query Language.

VPN: Virtual Private Network.

W2K: Windows 2000.

WEP: Wired Equivalent Privacy.

WLAN: wireless local area network.

## Competing interests

The author(s) declare that they have no competing interests.

## Authors' contributions

DC, the principal investigator, conducted most of the experiments. HFO was the sponsor of a fellowship awarded to DC by the National Library of Medicine, NIH of the US. SMS was an advisor for genome-related information search. They contributed to the design, coordination, support, and performing of experiments.

## Pre-publication history

The pre-publication history for this paper can be accessed here:



## References

[B1] Chen D, Soong SJ, Grimes GJ, Orthner HF (2004). Wireless local area network in a prehospital environment. BMC Med Inform Decis Mak.

[B2] Hersh W, Price S, Donohoe L (2000). Assessing thesaurus-based query expansion using the UMLS Metathesaurus. Proc AMIA Symp.

[B3] Hersh WR, Hickam DH (1992). A comparison of retrieval effectiveness for three methods of indexing medical literature. Am J Med Sci.

[B4] Hersh WR, Hickam DH (1993). A comparison of two methods for indexing and retrieval from a full-text medical database. Med Decis Making.

[B5] Hersh WR, Hickam DH, Haynes RB, McKibbon KA (1994). A performance and failure analysis of SAPHIRE with a MEDLINE test collection. J Am Med Inform Assoc.

[B6] Nikita Borisov IG, David Wagner (1999). Intercepting Mobile Communications: The Insecurity of 802.11. http://www.isaac.cs.berkeley.edu/isaac/wep-draft.pdf.

[B7] Kagolovsky Y, Moehr JR (2003). Terminological problems in information retrieval. J Med Syst.

[B8] Kagolovsky Y, Moehr JR (2003). Current status of the evaluation of information retrieval. J Med Syst.

[B9] Kagolovsky Y, Moehr JR (2004). Introducing a conceptual information retrieval (IR) framework. J Med Syst.

[B10] Kagolovsky Y, Moehr JR (2004). A new look at information retrieval evaluation: proposal for solutions. J Med Syst.

[B11] Nenadic G, Mima H, Spasic I, Ananiadou S, Tsujii J (2002). Terminology-driven literature mining and knowledge acquisition in biomedicine. Int J Med Inform.

[B12] Hong X, Harris CJ, Chen S, Nenadic G, Mima H, Spasic I, Ananiadou S, Tsujii J (2004). Robust neurofuzzy rule base knowledge extraction and estimation using subspace decomposition combined with regularization and D-optimality Terminology-driven literature mining and knowledge acquisition in biomedicine. IEEE Trans Syst Man Cybern B Cybern.

[B13] Boyer C, Baujard O, Baujard V, Aurel S, Selby M, Appel RD (1997). Health On the Net automated database of health and medical information. Int J Med Inf.

[B14] Kuller AB, Wessel CB, Ginn DS, Martin TP (1993). Quality filtering of the clinical literature by librarians and physicians. Bull Med Libr Assoc.

[B15] Schleyer TK (1999). Clinical decision-making and the Internet. J Am Coll Dent.

[B16] Quintana Y (1998). Intelligent medical information filtering. Int J Med Inf.

[B17] Duncan RG, Shabot MM (2000). Secure remote access to a clinical data repository using a wireless personal digital assistant (PDA). Proc AMIA Symp.

[B18] Orthner HF, Scherrer JR, Dahlen R (1994). Sharing and communicating health care information: summary and recommendations. Int J Biomed Comput.

[B19] Georgiadis CK, Mavridis IK, Pangalos GI (2003). Healthcare teams over the Internet: programming a certificate-based approach. Int J Med Inf.

[B20] Schull H, Schmidt V (2000). MedStage – platform for information and communication in healthcare. Stud Health Technol Inform.

[B21] Lippoff O (2001). Wireless invasion: health care's evolution to wireless connectivity. J Med Pract Manage.

